# Combination Patterns of Major *R* Genes Determine the Level of Resistance to the *M*. *oryzae* in Rice (*Oryza sativa* L.)

**DOI:** 10.1371/journal.pone.0126130

**Published:** 2015-06-01

**Authors:** Yunyu Wu, Ning Xiao, Ling Yu, Cunhong Pan, Yuhong Li, Xiaoxiang Zhang, Guangqing Liu, Zhengyuan Dai, Xuebiao Pan, Aihong Li

**Affiliations:** 1 Lixiahe Agricultural Research Institute of Jiangsu Province, Yangzhou, 225007, P.R. China; 2 Key Laboratory of Plant Functional Genomics, Ministry of Education, Yangzhou University, Yangzhou, 225009, P.R. China; China National Rice Research Institute, CHINA

## Abstract

Rice blast caused by *Magnaporthe oryzae* is the most devastating disease of rice and poses a serious threat to world food security. In this study, the distribution and effectiveness of 18 *R* genes in 277 accessions were investigated based on pathogenicity assays and molecular markers. The results showed that most of the accessions exhibited some degree of resistance (resistance frequency, RF >50%). Accordingly, most of the accessions were observed to harbor two or more *R* genes, and the number of *R* genes harbored in accessions was significantly positively correlated with RF. Some *R* genes were demonstrated to be specifically distributed in the genomes of rice sub-species, such as *Pigm*, *Pi9*, *Pi5* and *Pi1*, which were only detected in *indica*-type accessions, and *Pik* and *Piz*, which were just harbored in *japonica*-type accessions. By analyzing the relationship between *R* genes and RF using a multiple stepwise regression model, the *R* genes *Pid3*, *Pi5*, *Pi9*, *Pi54*, *Pigm *and *Pit* were found to show the main effects against *M*. *oryzae* in *indica*-type accessions, while *Pita*, *Pb1*, *Pik*, *Pizt* and *Pia* were indicated to exhibit the main effects against *M*. *oryzae* in *japonica*-type accessions. Principal component analysis (PCA) and cluster analysis revealed that combination patterns of major *R* genes were the main factors determining the resistance of rice varieties to *M*. *oryzae*, such as ‘*Pi9*+*Pi54*’, ‘*Pid3*+*Pigm*’, ‘*Pi5*+*Pid3*+*Pigm*’, ‘*Pi5*+*Pi54*+*Pid3*+*Pigm*’, ‘*Pi5*+*Pid3*’ and ‘*Pi5*+*Pit*+*Pid3*’ in *indica*-type accessions and ‘*Pik+Pib*’, ‘*Pik+Pita*’, ‘*Pik+Pb1*’, ‘*Pizt+Pia*’ and ‘*Pizt+Pita*’ in *japonica*-type accessions, which were able to confer effective resistance against *M*. *oryzae*. The above results provide good theoretical support for the rational utilization of combinations of major *R* genes in developing rice cultivars with broad-spectrum resistance.

## Introduction

Rice (*Oryza sativa* L.) is one of the major food crops worldwide, feeding more than half of the world’s population [1]. Rice blast caused by the fungal pathogen *Magnaporthe oryzae* is the most severe disease of rice worldwide. Due to its widespread distribution and ability to survive in a wide range of environmental conditions, yield losses between 10% and 30%, i.e. destroying rice that could have fed more than 60 million people [2]. Over the last 30 years, there were three major blast epidemics occurred in China during 1982–1985, 1992–1994, and 2001–2005 [3]. The development of resistant cultivars via the introduction of major *R* genes into elite rice varieties is the most economical and environmentally friendly way to protect crops against this disease [2]. Therefore, identification and characterization of blast resistance genes in germplasm collections is critical for rice improvement.

To date, more than 100 rice blast resistance genes have been identified in rice [4,5], and 20 major *R* genes (*Pi1*, *Pi2*, *Pi5*, *Pi9*, *Pid3*, *Pi25*, *Pi36*, *Pi37*, *Pi54*, *Pia*/*Pi-Co39*, *Pib*, *Pid2*, *Pikm*, *Pikp*, *Pik*, *Pish*, *Pit*, *Pita* and *Pizt*) [6–10] and two partial resistance genes (*pi21* and *Pb1*) [11,12] have been cloned and characterized. All of the cloned *R* genes (except *Pid2* and *pi21*) belong to the nucleotide-binding site and leucine-rich repeat (NBS–LRR) class of *R* genes [13], and some of these *R* genes such as *Pi5*, *Pia/Pi-Co39*, *Pikm*, *Pik* and *Pikp* require two adjacent NBS-LRR class genes for full functionality [7,8,14]. However, *Pid2* encodes a B-lectin receptor kinase [15], while *pi21* encodes a proline-rich protein that containing a putative heavy metal-binding domain and putative protein-protein interaction motifs [11]. Although characterization of these *R* genes has advanced our understanding of the molecular basis of blast resistance in rice, we know little about how these cloned *R* genes are distributed in modern cultivated rice varieties. Additionally, information both on the resistance effects of *R* genes in different genetic backgrounds and on which genes are more effective in breeding practice is also missing. Furthermore, there are more than 100 *R* genes in the rice genome, which means that a single germplasm may harbor several *R* genes in various combinations, but the fact that resistance reactions show significant differences between germplasms indicates that these variations could be caused by different *R* gene combinations. Therefore, the question arises of which *R* genes could be combined to provide a favorable resistance effect across multiple backgrounds. Addressing this question will be helpful in the improvement of blast resistance breeding programs.

Accurate identification of a particular *R* gene in diverse elite germplasm is the first step for utilization of *R* genes in rice breeding programs. The conventional methodology to identify allelic variation based on phenotype is limited by large workload and time-consuming, as well as the strong dependence on environmental conditions. However, for *R* genes that have been isolated, it is now possible to replace phenotypes with molecular markers as the basis for defining alleles [10]. Over the last several decades, many PCR-based tightly linked markers (LMs) have been developed closely associated with a number of *R* genes, such as *Pi1* [16], *Pi2* [17], *Pi5* [18], *Pi9* [19]. These LMs offer an efficient and rapid way to select for the presence of target *R* genes in gene introgression and gene pyramiding. Moreover, the identification of functional markers (FMs), which include single nucleotide polymorphisms (SNPs) and insertion/deletions (InDels), were derived from polymorphic sites within genes causally involved in phenotypic trait variation and is particularly useful in several genetic backgrounds [20]. Some FMs specific to *R* genes, such as *Pita* [21], *Pib* [22], *Pit* [23] and *Pid3* [24] have been developed, which will provide convenient ways to identify target genes.

In the present study, the distribution of 18 *R* genes was analyzed in 277 accessions of Chinese elite rice parental lines and the donors of *R* genes using FMs and LMs, and the resistance frequency (RF) in the 277 accessions was evaluated by a total of 76 isolates. The *R* genes showing the main effects to *M*. *oryzae* in *indica* and *japonica* rice genome were identified through multiple stepwise regression analysis. The combination patterns of major *R* genes in the *indica-*type and *japonica-*type rice germplasm were ascertained based on principal component analysis (PCA) and cluster analysis of various gene combination factors. The results of this study are expected to guide the deployment and combination of *R* genes and provide useful information to improve blast resistance.

## Materials and Methods

### Plant materials

A total of 277 accessions (128 *japonica* and 149 *indica*) mainly consisting of Chinese elite rice parental lines, were analyzed in the present study (S1 Table). Most of the accessions used in this study were categorized as showing good agronomic traits, eating quality, yield traits, grain quality and blast resistance and have been widely used as parental lines to develop new varieties in recent years. To perform a broad-scale investigation of *R* gene-based resistance, three sets of differential lines and the *R* gene donors were used as standard check varieties. The first of these sets was composed of Japanese differentials [25, 26], including Fukunishiki (*Piz*), Toride 1 (*Pizt*), K59 (*Pit*), Kanto 51 (*Pik*), Tsuyuake (*Pikm*), K60 (*Pikp*), BL 1 (*Pib*), Yashiromochi (Pita), K3 (*Pi54*), Shin 2 (*Piks*) and Pi No.4 (*Pita2*). The second set consisted of Chinese differentials, including Tetep (*Pi1*, *Pi5*, *Pi54*), Zhenglong 13 (unknown), Sifeng 43 (*Pia*, *Pib*), Dongnong 363 (*Pia*, *Pik*), Kanto 51 (*Pik*), Hejiang 18 (*Pia*) and Lijangxituanheigu (LTH); the third set consisted of IRRI differentials, including six resistant near isogenic lines (NILs) in the genetic background of Co39 [27]. For the *Pi9*, *Pi40*, *Pigm*, *Pid2* and *Pid3* genes, the donor lines 75-1-127 [28], IR65482-4-136-2-2 [29], Gumei 4 [30] and Digu [31] were employed in this study.

### Blast isolates, inoculation and disease evaluation

To evaluate blast resistance, a total of 76 isolates collected from Sichuan (SC), Anhui (AH), Jiangsu (JS), Hainan (HN), Hubei (HB), and Zhejiang (ZJ) provinces in China from 2010–2012 were examined in this study (S2 Table). The plants used for inoculation were grown in 60 cm×30 cm×4 cm plastic trays with sieved garden soil. Each tray contained 42 experimental materials and two highly susceptible cultivars, Co39 and LTH (susceptible control). A total of 7 trays were used for each blast isolate inoculation experiment, containing 277 lines each with at least 10 plants. Three-week-old rice seedlings were placed in inoculation chambers and inoculated by spraying with 40 ml of conidial suspensions (5×10^4^ conidia/ml) with 0.02% Tween-20 as described by Huang et al. [32]. The inoculated seedlings were kept in dark chambers at 26°C with 95–100% relative humidity for 24 h and then transferred to the greenhouse where they were grown under a 12 h light/12 h dark cycle and 90% relative humidity. The inoculated seedlings were maintained under these conditions for 7 days to allow disease development. A randomized complete block design with two replicates was applied to the entire inoculation experiment.

Disease reactions were assessed 7 days after inoculation, and lesions on the rice leaves were evaluated on a scale of 0 to 5 according to standard procedures [33], where 0 indicated no visible lesion; 1 indicated only small point lesions appearing at the sites of infections; 2 indicated lesion sizes smaller than 2 mm and no visible fungal mass; 3 indicated that lesions were greater than 2 mm occupying 10% or less of the leaf area; 4 indicates that lesions were greater than 3 mm occupying more than 10% and less than 50% of the leaf area; and 5 indicates that lesions were greater than 50% of the leaf area. Plants with scores of 0–2 were classified as resistant (R) and those with scores of 3–5 were classified as susceptible (S) [34]. The RF of the accessions in relation to the isolates of *M*. *oryzae* could be calculated according to the following formula: RF = (the number of incompatible isolates / the total number of isolates used) *100%. Each RF value was assessed from two technical replicates.

### DNA markers and genotyping of 277 rice germplasms

A total of 15 FMs and 9 LMs were selected from published primer sequences to detect 18 *R* genes, including six alleles of the Piz locus (*Piz*, *Pizt*, *Pi2*, *Pi9*, *Pi40* and *Pigm*) [35, 36], two alleles of the Pik locus (*Pik* and *Pi1*) [10, 37] and ten independent *R* genes (*Pia*, *Pib*, *Pit*, *Pish*, *Pi5*, *Pi54*, *Pita*, *Pid2*, *Pid3*, and *Pb1*) [6] in the 277 accessions. Detailed information on the markers is provided in Table 1.

**Table 1:** Information of molecular markers for rice blast resistance genes.

Chr.	Target genes	Marker name	Marker type	primer		Anneal temperature	Restriction enzyme	References
				Forward	Reverse			
1	*Pit*	tdDN	FM	GGAAAAATAGAGTCAAACCGCC	CCTTTCGATGTTTTTTCTATATAAGC	60		[23]
1		tdDK	FM	GTGCCACGTGTCGCCTTCCCGTTG	CCTTTCGATGTTTTTTCTATATAAGC	60		
1	*Pish*	RM6648	LM	GATCGATCATGGCCAGAGAG	ACAGCAGGTTGATGAGGACC	55		[38]
1		RM5811	LM	TTCGCGCTCTCCAAGCTC	GGATTTGGTCGAACAGGTTG	55		
2	*Pib*	Pibdom	FM	GAACAATGCCCAAACTTGAGA	GGGTCCACATGTCAGTGAGC	55		[22]
2	*pib*	Lys145	FM	TCGGTGCCTCGGTAGTCAGT	GGGAAGCGGATCCTAGGTCT	55		
6	*Pid3*	CAP3	FM	CCTCACGTTTCTACGTCTTG	CACACCATTTCTGATGAACC	60	Nde I	[24]
6	*Pigm*	S29742	LM	CAGTGAAACGAACGCTATG	AATAGGAAGGGTTGATGTTG	56		[30]
6		ZJ58.7–6	LM	ACTTGCTGGGAGAAGGATT	AGTTCGTACTTTTCAGGCT	55		[39]
6	*Pi9*	F9	FM	TGATTATGTTTTTTATGTGGGG	ATTAGTGAGATCCATTGTTCC	55		[28]
6	*Pi9/Piz*	Pi9-2	FM	GGAGAAAATACACCCGACATGTGACG	CGGCTCGAAAACGAACGTACCATT	55		[19]
6	*Pizt/Pi2*	M-Pi2	FM	CAGCGATGGTATGAGCACAA	CGTTCCTATACTGCCACATCG	55		[40]
6	*Pizt*	R2123	LM	GGTGCCTCCTTCCATATGCCAGCA	TGCAGCACAAGTTATCAGCTTGGCGACT	60	Hinf I	[19]
6	*Pi2*	AP22	LM	GTGCATGAGTCCAGCTCAAA	GTGTACTCCCATGGCTGCTC	55		[17]
	*Pi40*	Pi40-509	LM	CAACAAACGGGTCGACAAAGG	CCCCCAGGTCGTGATACCTTC	58	Tsp509 I	[29]
6	*Pid2*	Pid2A	FM	CTTTTGTACTGAGGGACCAC	CGATTATCTCAAGCAAAACC	55	Mlu I	[15]
9	*Pi5*	JJ817	FM	GATATGGTTGAAAAGCTAATCTCA	ATCATTGTCCTTCATATTCAGAGT	60		[18]
11	*Pia*	Pia-STS	FM	CTTTTGAGCTTGATTGGTCTGC	CTATTGCACCAGAGGGACCAG	65		[8]
11	*Pi54*	Pikh-1	FM	CAATCTCCAAAGTTTTCAGG	GCTTCAATCACTGCTAGACC	55		[41]
11	*Pik*	dCAPS-2953	FM	TTCGAGGCCCTACCAAGACA	CATGGAAGGCTATCCTTGGTA	60	Kpn I	[37]
11	*Pi1*	Rm224	LM	ATCGATCGATCTTCACGAGG	TGCTATAAAAGGCATTCGGG	55		[16]
11	*Pb1*	RM26998	LM	ACGCACGCACATCCTCTTCC	CGGTTCTCCATCTGAAATCCCTAGC	55		[12]
12	*Pita*	YL155 /YL87	FM	AGCAGGTTATAAGCTAGGCC	CTACCAACAAGTTCATCAAA	60		[21]
12	*pita*	YL183/YL87	FM	AGCAGGTTATAAGCTAGCTAT	CTACCAACAAGTTCATCAAA	60		

PCR amplification was conducted following a standard protocol [42], including pre-denaturation for 5 min at 94°C, followed by 35 cycles of 45 s at 94°C, 45 s at the annealing temperature indicated in Table 1 and 1 min at 72°C, followed by a final extension at 72°C for 10 min. For dCAPS marker analysis, the PCR products were digested following the manufacturer’s instructions. DNA bands were visualized on an 8% denaturing polyacrylamide gel or a 4% agarose gel according to their relative fragment sizes. The reactions that amplified the same bands as the target gene donors were labeled with a “+” sign, and the others were labeled with a “-” sign.

### Data analysis

The relationship between *R* genes and RF was analyzed using a multiple stepwise regression model, which is an available option in Matlab (V.7.0) software, according to Xu et al. [43]. *R* genes that were significantly correlated with RF (*P*≤0.01) were subjected to various *R* gene combination factors according to factor analysis (principal component analysis, PCA) in SPSS (V.21) software. Based on various *R* gene combination factors, the germplasms were clustered into several groups. Each group represents one class of *R* gene combination patterns found among the germplasms. Significant differences in the RF between population groups were analyzed by one-way analysis of variance (ANOVA) [44]

## Results

### Genotypic assays for 18 *R* genes in the rice germplasms

**Fig 1:**
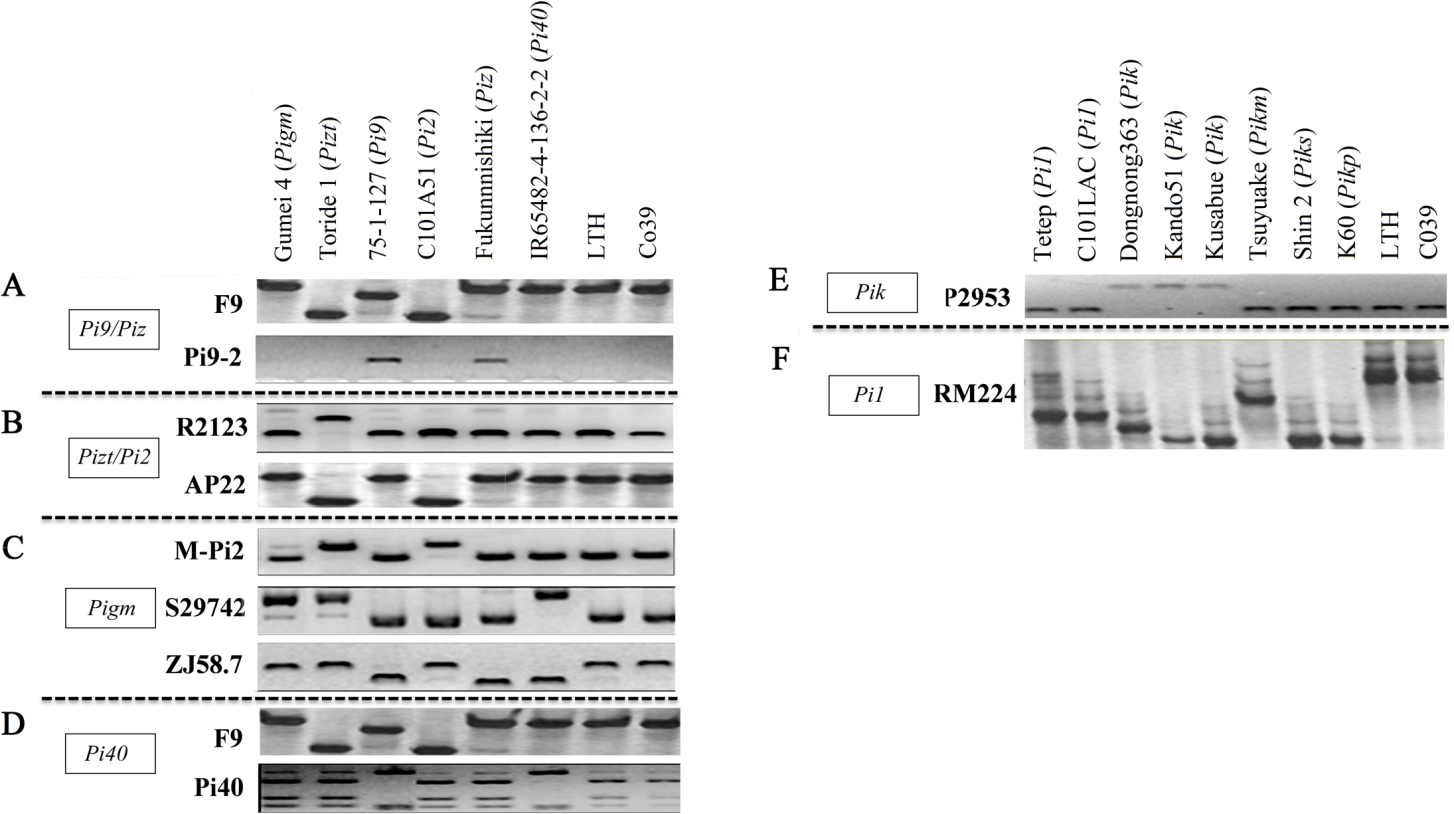
PCR amplification patterns of molecular markers that discriminate each of the *R* genes at the *Piz* and *Pik* loci.

Ten independent *R* genes, including *Pia*, *Pib*, *Pit*, *Pish*, *Pi5*, *Pi54*, *Pita*, *Pid2*, *Pid3*, and *Pb1*, were easily identified based on their FMs or LMs (S1 Fig). However, the alleles of complex gene loci were difficult to distinguish from each other because of their sequence similarity. To distinguish the six alleles of the Piz locus (*Pi2*, *Pi9*, *Pizt*, *Piz*, *Pigm* and *Pi40*) from each other, several markers were selected from published primer sequences. An InDel polymorphism specific for *Pi9* donor 75-1-127 was obtained using the marker F9, which discriminates Pi9 from other alleles (Fig 1A). The Pi9-2 is known to detect *Pi9-* and *Piz-*containing genotypes in a previous study [19], indicating its suitability as a *Pi9-* and *Piz-*specific marker. Therefore, the combination of F9 and Pi9-2 can distinguish *Pi9* and *Piz* from other alleles. The dCAPS marker R2123 revealed a polymorphism between the *Pizt* donor Toride 1 and the other tested genotypes [19], thus behaving as a *Pizt-*specific marker in this study. In addition, a *Pi2*-selective marker [39], AP22, detected a polymorphism between the *Pi2*/*Pizt* donors and the other tested genotypes. Thus, these two markers are able to differentiate *Pi2* and *Pizt* from other alleles (Fig 1B). To assess *Pigm* in the Chinese elite rice parental lines, two co-segregating markers S29742 and ZJ58.7 were used in the present study [30, 39], but the detection results showed that they could not distinguish *Pigm* from *Pizt*; hence, the InDel marker M-Pi2 was introduced to detect *Pigm*, together with S29742 and ZJ58.7. The results revealed that the combined use of these three markers could distinguish *Pigm* from other alleles (Fig 1C). Similar to *Pigm*, the dCAPS marker Pi40 was not able to discriminate between *Pi40* and the other alleles; therefore, the marker F9 was used to detect *Pi40* together with the Pi40 marker (Fig 1D). Another multiple gene complex loci is the *Pik* locus on chromosome 11, which consisted of *Pi1*, *Pik*, *Pikm*, *Pikp* and *Piks* alleles. In this study, only *Pi1* and *Pik* were detected for breeding practice purpose. The dCAPS marker 2953 was verified to be a specific marker for *Pik* [37] and was further confirmed to distinguish *Pik* from various other *Pik* alleles in this study (Fig 1E). The marker RM224 has been reported to be linked to *Pi1* [16] and was further demonstrated to be a Pi1-specific marker in this study (Fig 1F).

### The number of *R* genes present in the accessions is positively correlated with the RF against *M*. *oryzae* in the Chinese elite rice parental lines

**Fig 2:**
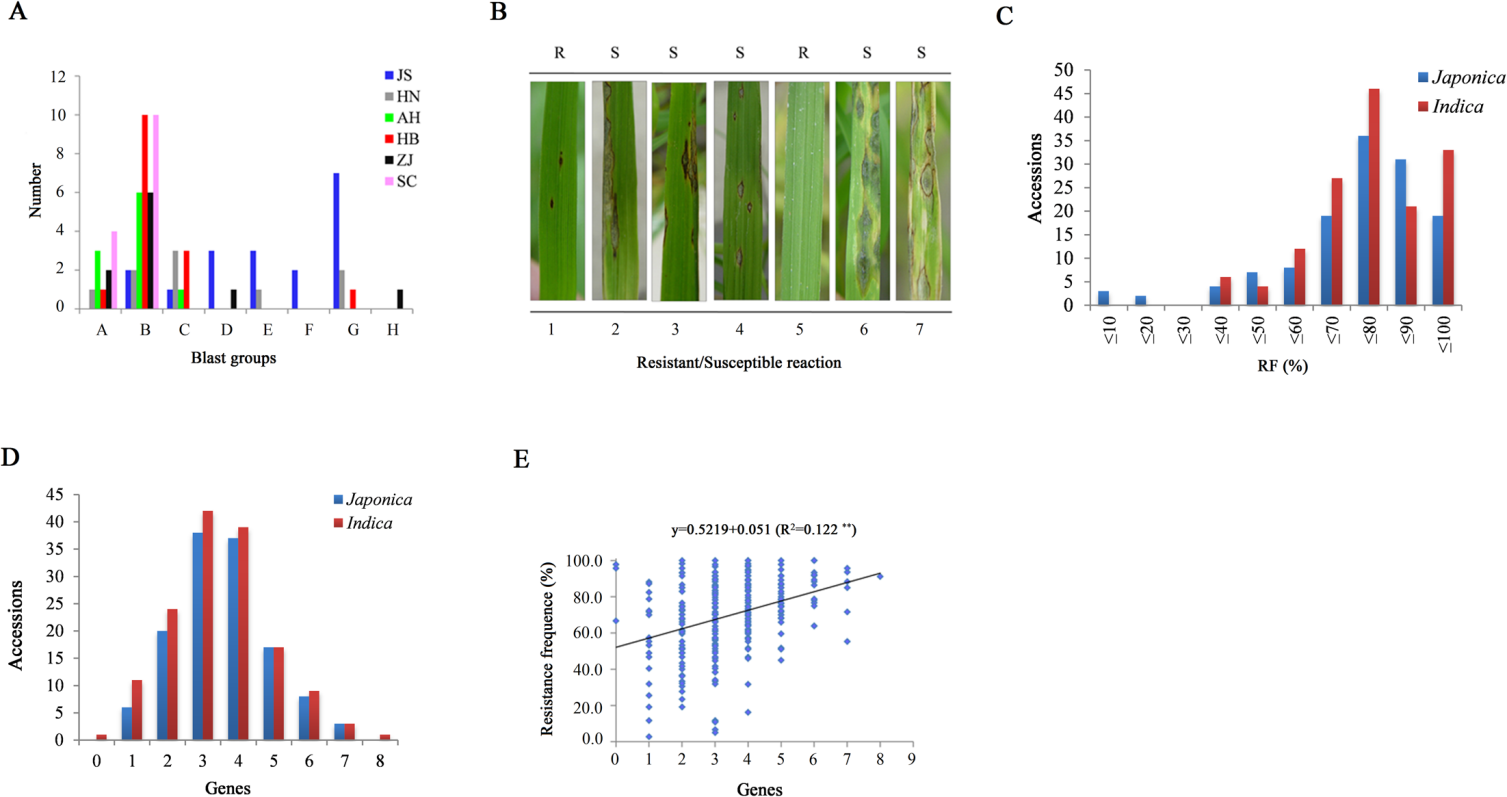
The disease reaction patterns in relation to the *M*. *oryzae* and 18 *R* genes detection in 277 rice accessions. A. The distribution of isolates in 6 typical provinces. B. The disease reactions of a typical blast isolate to 7 Chinese differential rice cultivars, 1–7 represent Tetep, Zhenlong 13, Sifeng 43, Dongnong 363, Kano51, Hejiang 18 and LTH, respectively. C. The distribution of RF values among accessions tested. D. The number of *R* genes present in 277 rice accessions. E. The relationship between the number of *R* genes and RF values.

According to the disease reactions to 7 Chinese differential rice cultivars, the 76 blast isolates obtained from rice blast specimens collected from 6 provinces were allocated to 8 groups and 24 races (Fig 2A), These results indicated that the isolates were highly diverse, with resistance/susceptibility being observed among the differential rice cultivars (Fig 2B). Therefore, the isolates could be used as differential strains in this study, with the exception of 176-1-1 (ZH), which showed avirulence on all of the differential rice cultivars (S3 Table). The inoculation results revealed that the accessions exhibited significant difierences in their reaction patterns and degrees of susceptibility to the isolates. We further calculated the RF of each accession and found that the values exhibited a skewed distribution that mostly ranged from 50–100% (Fig 2C), implying that most of the accessions showed some degree of resistance to *M*. *oryzae*.

Most of the accessions harbored more than one of the 18 detected *R* genes, except for three *indica*-type accessions (S1 Table). The number of *R* genes found in the accessions was normally distributed, mostly ranging from 2–5 genes (Fig 2D). Compared with the results of the pathogenicity assays, we observed that the number of *R* genes present in the accessions was significantly positively correlated with the RF (Fig 2E; R^2^ = 0.122, P<0.01), which means that the greater the number of R genes found in the accessions, the higher the RF against *M*. *oryzae*.

### Various *R* genes show the main effects against *M*. *oryzae* in the *indica* and *japonica* rice genomes

**Fig 3:**
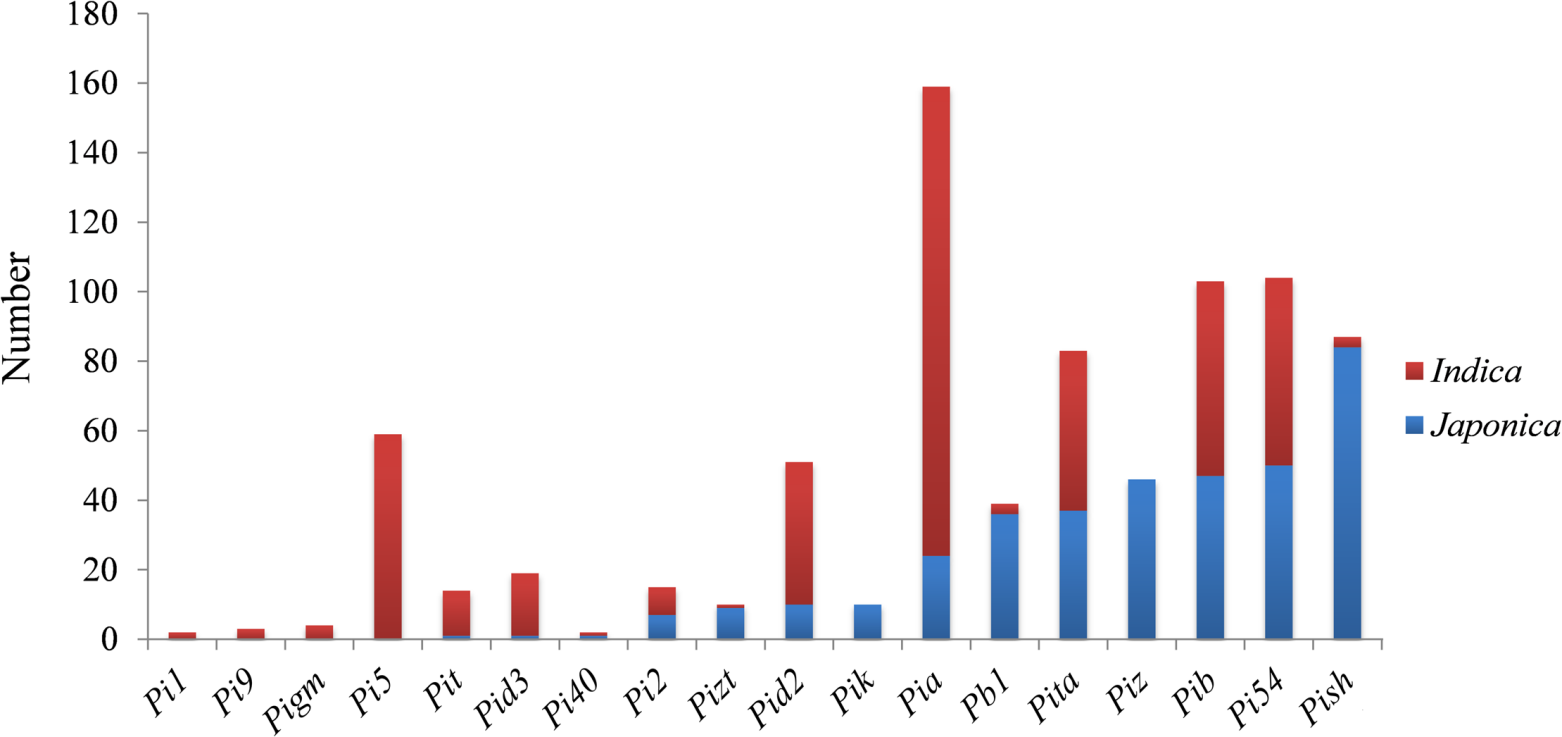
The distribution of *R* genes detected in 277 rice accessions.

A total of 18 *R* genes were detected in 277 accessions using a set of 24 FMs and LMs, and the results demonstrated that most of these *R* genes were present in both the *indica* and *japonica* sub-species genomes. With the exception of the *R* genes *Pi2*, *Pi40*, *Pita*, *Pib* and *Pi54* which were evenly distributed in the *indica*-*japonica* accessions, the other *R* genes were found to show a disequilibrium distribution between the *indica* and *japonica* sub-species genomes. For example, the *R* genes *Pigm*, *Pi9*, *Pi5* and *Pi1* were only found in *indica*-type accessions, and *Pik* and *Piz* were just present in *japonica*-type accessions. The distribution frequency of the *R* genes *Pit*, *Pid2*, *Pid3* and *Pia* was higher in *indica*-type accessions than in *japonica*-type accessions, but the distribution frequency of the *R* genes *Pizt*, *Pb1* and *Pish* was higher in *japonica*-type accessions than in *indica*-type accessions (Fig 3 and S4 Table). This distribution specificity of the *R* genes implies that the *R* genes conferring resistance to *M*. *oryzae* may differ between the *indica* and *japonica* sub-species genomes.

By analyzing the relationship between the *R* genes and RF using a multiple stepwise regression model, the *R* genes *Pid3*, *Pi5*, *Pi9*, *Pi54*, *Pigm* and *Pit* were found to show the main effects against *M*. *oryzae* in *indica*-type accessions, together accounting for 44.8% (R^2^ = 44.8%, P<0.01) of the observed phenotypic variance, with partial correlation coefficients of 0.273, 0.271, 0.194, 0.190, 0.171 and 0.166, respectively (Table 2). In contrast to the results for the *indica*-type accessions, the *R* genes *Pita*, *Pb1*, *Pik*, *Pizt* and *Pia* were indicated to show the main effects against *M*. *oryzae* in *japonica*-type accessions, which explained 36.2% (R^2^ = 36.2%, P<0.01) of the observed phenotypic variance overall, with partial correlation coefficients of 0.468, 0.384, 0.268, 0.243 and 0.197, respectively (Table 2). In addition, we found that many of the *R* genes were associated with a province-specific resistance in the various rice-growing regions of China, such as *Pi5* was detected to explain 21.0% phenotypic variances observed relative to isolates originating from HN Provence in *indica*-type accessions (S5 Table). The above results imply that certain *R* genes showed the main effects of the resistance to *M*. *oryzae*, and the resistance and susceptibility of the accessions, determined by their *R* genes, varied between the *indica* and *japonica* sub-species backgrounds.

**Table 2:** The effect of rice blast resistance genes against *M*. *oryzae*.

	Resistance frequency (%)	Gene type																			R^2^
		*Pit*	*Pish*	*Pib*	*Pid3*	*Pid2*	*Pigm*	*Pi2*	*Pi9*	*Piz*	*Pizt*	*Pi40*	*Pi5*	*Pia*	*Pi54*	*Pik*	*Pi1*	*Pb1*	*Pita*		
*indica*	Total	0.166			0.273		0.171		0.194				0.271		0.190					0.448**	
*japonica*	Total										0.243			0.197		0.268		0.384	0.468	0.362*	

*:P = 0.05

**:P = 0.01

### *R* gene combination patterns are the main factors determining the resistance of rice varieties to *M*. *oryzae*

**Fig 4:**
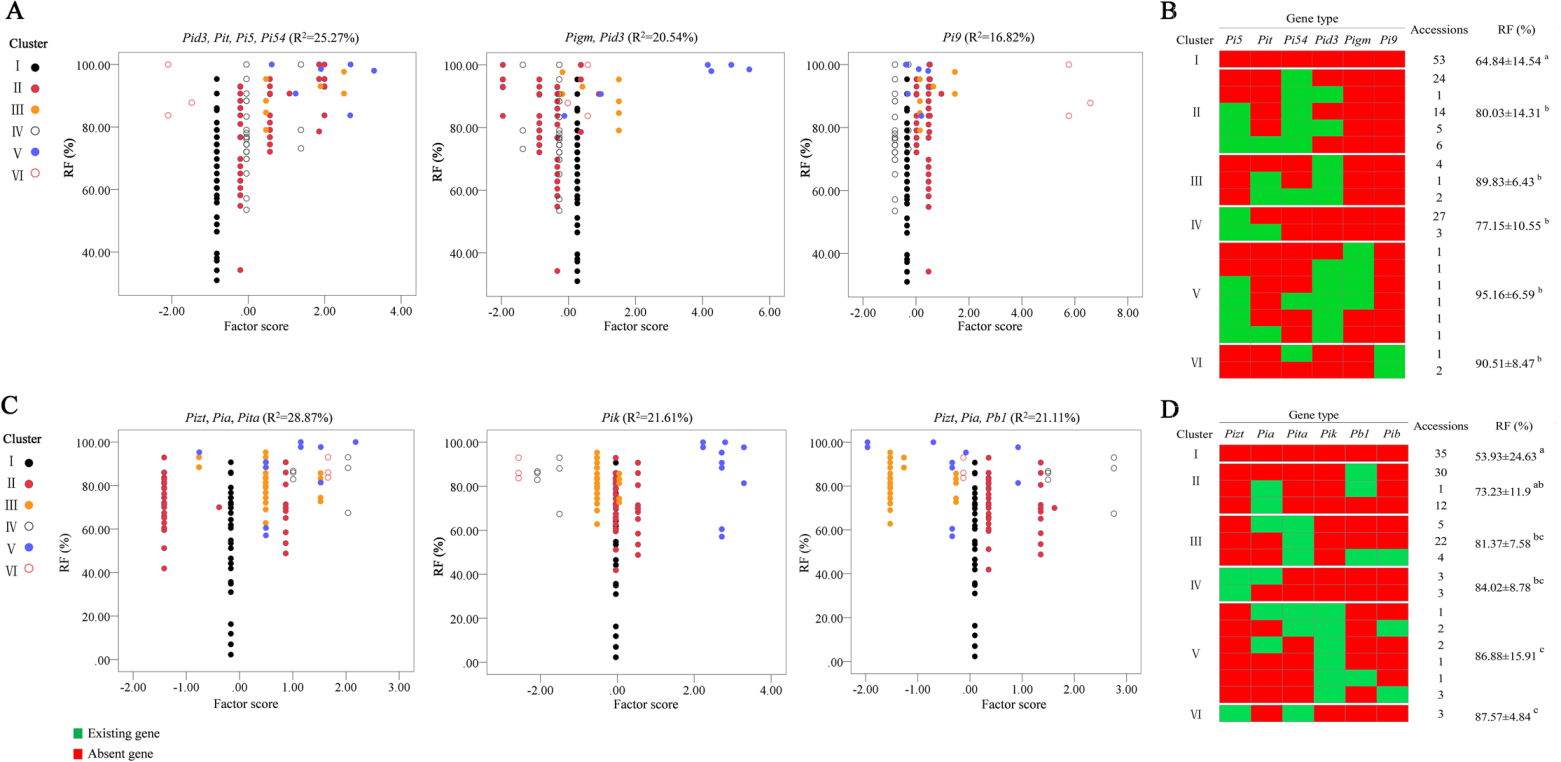
*R* gene combination factors and gene clusters in *indica*-type and *japonica*-type accessions. A. *R* gene combination factors in *indica*-type accessions. B. Gene clusters in *indica*-type accessions. C. *R* gene combination factors in *japonica*-type accessions. D. Gene clusters in *japonica*-type accessions.

The genes that showed the main effects against *M*. *oryzae* in *indica*-type accessions were classified into three types of *R* gene combination factors: ‘*Pid3*, *Pi5*, *Pit*, *Pi54*’ (R^2^ = 25.27%), ‘*Pid3*, *Pigm*’ (R^2^ = 20.54%) ‘*Pi9*’ (R^2^ = 16.82%) and, according to the principal component analysis results (PCA, KMO = 0.65, *P* = 0.01), and the 149 *indica* accessions were further categorized into 6 groups based on cluster analysis with *R* gene combination factors (Fig 4A). Cluster I did not include any of these *R* gene combination factors and presented an RF of 64.84% (Fig 4B). Clusters II, III and IV mainly contained *R* gene combination factors (*Pid3*, *Pi5*, *Pit* and *Pi54*) and showed RF values of 80.03%, 89.83% and 77.15% respectively. Cluster V was mainly composed of *R* gene combination factors (*Pid3* and *Pigm*) and exhibited RF values of 95.16%. Cluster VI included the *R* gene combination factor *Pi9* and showed an RF of 90.51%. The RF values of clusters II, III, IV, Vand VI were significantly higher than that of cluster I, suggesting that major *R* genes could significantly improve the resistance of the rice germplasm to *M*. *oryzae* (P<0.01). Furthermore, the results showed that there were significant differences in the level of resistance between clusters that contained various *R* gene combination factors. For example, cluster II mainly contained *R* gene combination factors (*Pid3*, *Pi5*, *Pit* and *Pi54*) and could be combined into 5 gene combination patterns (*Pi54*, *Pi54*+*Pid3*, *Pi5*+*Pi54*, *Pi5*+*Pi54*+*Pid3* and *Pi5*+*Pit*+*Pi54*). However, the RF of cluster II was significantly lower than that of cluster VI, which contained the *R* gene factor ‘*Pi9*’ with the combination patterns ‘*Pi9*+*Pi54*’ and ‘*Pi9*’, indicating that the *R* gene combination pattern may be the main factor determining rice variety resistance to *M*. *oryzae* in *indica*-background accessions.

Similarly, three *R* gene combination factors, ‘*Pizt*, *Pia*, *Pita*’(R^2^ = 28.87%), ‘*Pik*’ (R^2^ = 21.61%) and ‘*Pizt*, *Pia*, *Pb1*’ (R^2^ = 21.11%), were categorized in *japonica*-type accessions among the *R* genes that showed the main effects against *M*. *oryzae* (KMO = 0.73, *P* = 0.01), and the 128 *japonica*-type accessions were further classified into six major groups, clusters I, II, III, IV, V and VI, based on the observed *R* gene combination factors (Fig 4C). Significant differences in the reaction patterns against *M*. *oryzae* were also observed among these cluster groups (Fig 4D). Cluster I did not include any of the above three *R* gene combination factors and presented an RF of only 53.93%. In contrast, clusters III and IV comprised *R* gene combination factors (*Pizt*, *Pia* and *Pita*) and showed RF values of 81.37% and 84.02%, respectively. Cluster V mainly included the *R* gene combination factor *Pik*, and its RF was 86.88%. Clusters II and VI mainly included *R* gene combination factors (*Pizt*, *Pia* and *Pb1*) and displayed RF values of 73.23% and 87.57%, respectively. The RF values of clusters III, IV, V and VI were significantly increased compared with that of cluster I (P<0.01). The resistance of cluster IV, carrying the ‘*Pizt+Pia*’ gene combination, and cluster VI, pyramiding ‘*Pizt+Pita*,’ was better than that of cluster II, carrying ‘*Pia+Pb1*,’ implying that various major *R* gene combination patterns resulted in different RFs in *japonica*-background accessions.

## Discussion

A large number of *R* genes have been identified and mapped in a variety of rice genotypes in previous works. Unfortunately, not all *R* genes are effective against pathogenic isolates of *M*. *oryzae*. Therefore, how to employ a few of these *R* genes to achieve broad-spectrum and durable resistance must be considered during rice blast resistance breeding.

According to the detection results of 18 *R* genes in 277 germplasms, we found that the distribution of *R* genes was significantly different between the *indica* and *japonica* sub-species genomes. For example, the *R* genes *Pit*, *Pid3*, *Pid2* and *Pia* were mainly detected in *indica*-type accessions, and *Pigm*, *Pi9*, *Pi5* and *Pi1* were only present in *indica*-type accessions. However, the *R* genes *Pizt*, *Pb1* and *Pish* were mainly identified in *japonica*-type accessions, and *Pik* and *Piz* were restricted to *japonica*-type accessions. These results were possible because these *R* genes have experienced significant differentiation due to reproductive isolation and decreased interflow between *indica*-*japonica* sub-species during the evolutionary process. Thus, it may be an effective strategy to transfer these *R* genes within subspecies using marker-assisted selection (MAS) to create new resistant germplasms, for example, transferring *Pid3*, *Pigm*, *Pi1* and *Pi5*, which originated in the *indica* genome, to the *japonica* rice genome.

The specific distribution of *R* genes also results in various *R* genes showing the main effects against *M*. *oryzae* between *indica* and *japonica* germplasms, such as the *R* genes *Pid3*, *Pi5*, *Pi9*, *Pi54*, *Pigm* and *Pit* in *indica*-type accessions and *Pita*, *Pb1*, *Pik*, *Pizt* and *Pia* in *japonica*-type accessions (Table 2). Additionally, we found that particular *R* genes were effective against isolates originating from particular regions of China, and these genes may therefore be specifically and effectively used in the target regions, such as *Pi2* in ZJ and HN Provinces and *Pib* in JS Province (S5 Table). Moreover, we noted that some *R* genes were distributed evenly between the *indica* and *japonica* genomes, such as *Pita* and *Pi54*. In contrast, *Pi54* was only found to show a main effect in *indica* backgrounds, and *Pita* only played a major role in *japonica* backgrounds (Table 2). These results indicated that some *R* genes like *Pita* and *Pi54* may be exhibiting a great interaction effect with the *indica*-*japonica* genome background. Therefore, the genetic background must be considered when using these *R* genes for breeding cultivars with broad-spectrum resistance.

It is thought to be a useful strategy to pyramid several *R* genes for breeding cultivars with broad-spectrum and durable resistance. However, random pyramiding of various *R* genes may not show an additive effect, as indicated by the combination of the *Piz5* and *Pita* genes in Co39 [45]. He et al. [46] also reported that the resistance effect of pyramiding lines with *Pi4* and *Pi1* was weaker than that in lines with the single genes in the Co39 background. Therefore, the question arises regarding which combination patterns of *R* genes are the most effective for achieving blast resistance.

In the present study, we demonstrated that *R* gene combination factor *Pi9* with combination patterns ‘*Pi9*+*Pi54*’ and ‘*Pi9*’, and the *R* gene combination factors ‘*Pid3*, *Pigm*’ with combination patterns ‘*Pid3*+*Pigm*’, ‘*Pi5*+*Pid3*+*Pigm*’, ‘*Pi5*+*Pi54*+*Pid3*+*Pigm*’, ‘*Pi5*+*Pid3*’ and ‘*Pi5*+*Pit*+*Pid3*’, could confer effective resistance to *M*. *oryzae* in *indica*-type accessions (Fig 4B). In contrast, in *japonica*-type accessions, *R* gene combination factor *Pik* with ‘*Pik+Pib*’, ‘*Pik+Pita*’, ‘*Pik+Pb1*’ combination patterns, *R* gene combination factors ‘*Pizt*, *Pia*, *Pita*’ with ‘*Pizt+Pia*’, ‘*Pizt+Pita*’ combination patterns, could confer effective resistance to *M*. *oryzae* (Fig 4D). These results indicated that combination patterns of major *R* genes might be the main factor determining the resistance of rice varieties to *M*. *oryzae*. Therefore, rational utilization of these gene combinations is essential for the development of elite rice cultivars with broad-spectrum resistance to the blast pathogen in target regions (S6 and S7 Tables).

Most of above-mentioned effective *R* gene combination patterns contained alleles of the *Piz* locus (*Pi9*, *Pigm* and *Pizt*). It has been shown that pyramiding lines with ‘*Pi1*+*Pi2*’ genes shows complete resistance to the most virulent races of the pathogen originating from southern China [16]. Furthermore, Yu et al. [39] demonstrated that the single *R* gene, *Pigm* and *Pi1*, possessed a complementary effect with respect to the blast isolates and the RF of pyramiding lines with ‘*Pigm+Pi1*’ to blast isolates originated from China was more than 90%. In addition, it has been documented that at least nine *R* genes *Pi1*, *Pi2*, *Pi5*, *Piz*, *Pi9*, *Pi40*, *Pizt*, *Pi33* and *Pigm* appear to confer broad-spectrum resistance to a number of isolates or races from one or more countries [20,34,47], and six of which are alleles of the *Piz* locus (*Pi2*, *Piz*, *Pi9*, *Pi40*, *Pizt* and *Pigm*). All of these results indicate that the alleles of the *Piz* locus play a key role in conferring resistance to *M*. *oryzae*. Therefore, it may be an effective strategy to use different alleles of *Piz* locus as the backbone for pyramiding with other *R* genes with strong complementary effect and broad-spectrum resistance, such as *Pi1*, *Pi5*, *Pi33*, *Pi54* and *Pid3*, could create new resistant germplasms and further enhance of the level and broad-spectrum of blast resistance in rice.

## Supporting Information

S1 Fig Molecular screening of molecular markers for 10 independent *R* genes. (TIF)

S1 Table Information of each of the 277 rice accessions, and their haplotype with respect to 18 *R* genes isolated. (XLSX)

S2 Table Information of blast populations for pathogenicity assays. (XLSX)

S3 Table Resistance reaction of 32 standard check varieties to 76 isolates of *M*. *oryzae*. (XLSX)

S4 Table Distribution of 18 rice blast resistance genes in *indica* and *japonica* genome. (XLSX)

S5 Table Effect of rice blast resistance genes against *M*. *oryzae* originated from six provinces, China. (XLSX)

S6 Table RF values of clusters in *indica*-type accessions in relation to *M*. *oryzae* originating from six provinces, China. (XLSX)

S7 Table RF values of clusters in *japonica*-type accessions in relation to *M*. *oryzae* originating from six provinces, China. (XLSX)
