# Supplementary figures and images for: Combination Patterns of Major R Genes Determine the Level of Resistance to the M. oryzae in Rice (Oryza sativa L.)
Source: PLoS One. 2015 Jun 1;10(6):e0126130. doi: 10.1371/journal.pone.0126130 (PMC4452627; doi:10.1371/journal.pone.0126130)

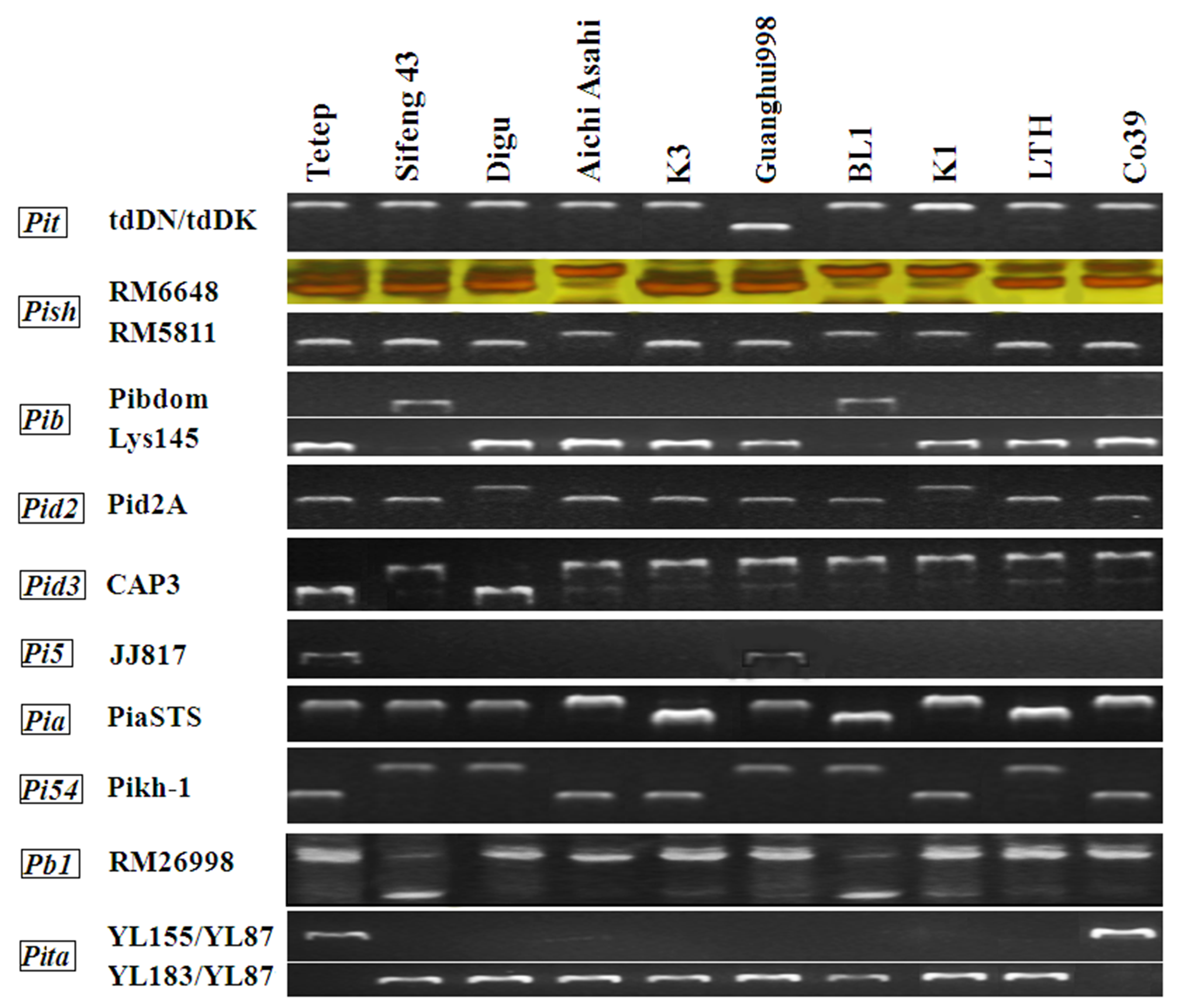

Supplement: S1 Fig — (TIF) [file pone.0126130.s001.tif]
